# DUSP5 suppresses interleukin-1β-induced chondrocyte inflammation and ameliorates osteoarthritis in rats

**DOI:** 10.18632/aging.202252

**Published:** 2020-12-15

**Authors:** Zhipeng Wu, Langhai Xu, Yuzhe He, Kai Xu, Zhonggai Chen, Safwat Adel Abdo Moqbel, Chiyuan Ma, Lifeng Jiang, Jisheng Ran, Lidong Wu, Ying Zhong

**Affiliations:** 1 Department of Orthopaedics, The First Affiliated Hospital of Zhejiang Chinese Medical University, Hangzhou, Zhejiang Province, China; 2 Department of Pain, Zhejiang Provincial People's Hospital, People's Hospital of Hangzhou Medical College, Hangzhou, China; 3 Department of Orthopedic Surgery, The Second Affiliated Hospital, Zhejiang University School of Medicine, Hangzhou, Zhejiang, China

**Keywords:** osteoarthritis, DUSP5, inflammation, NF-κB, ERK

## Abstract

Osteoarthritis (OA) is a chronic degenerative joint disease characterized by deterioration of articular cartilage. Dual specificity phosphatase 5 (DUSP5), a member of the DUSP subfamily, is known to regulate cellular inflammation. Here, we studied the relationship between DUSP5 and OA by knockdown and overexpression DUSP5, respectively. Results from *in vitro* experiments demonstrated that the knockdown of DUSP5 increased interleukin-1β (IL-1β)-induced expression of inflammatory genes, such as inducible nitric oxide synthase (iNOS), cyclooxygenase 2 (COX2), and matrix metalloproteinases (MMPs) in chondrocytes, whereas it decreased the expression of anti-inflammatory genes, such as tissue inhibitor of metalloproteinase 3 (TIMP3) and IL-10. Conversely, the overexpression of DUSP5 suppressed the IL-1β-induced expression of iNOS, COX-2, and MMPs, and upregulated the expression of TIMP3 and IL-10. Moreover, knockdown of DUSP5 enhanced the IL-1β-induced activation of NF-κB and ERK pathways, whereas its overexpression inhibited these pathways. DUSP5 overexpression prevented cartilage degeneration in a rat OA model, while its knockdown reversed that effect. Our findings reveal that DUSP5 suppresses IL-1β-induced chondrocyte inflammation by inhibiting the NF-κB and ERK signaling pathways and ameliorates OA.

## INTRODUCTION

Osteoarthritis (OA) is a common joint disease in the elderly population characterized by progressive extracellular matrix (ECM) destruction, synovial inflammation, articular cartilage degeneration, and subchondral bone changes [1–4]. Several factors, such as age, gender, genetics, and obesity, may increase the risk of developing OA [5, 6]. The treatment of OA has remained a challenge owing to its unknown pathogenesis [1, 7, 8]. Currently, the mainstay of OA treatment is to control the symptoms [2]. Conservative treatments for OA include steroids and non-steroidal anti-inflammatory drugs (NSAIDs). However, these are incapable of reducing the damage to the articular cartilage; moreover, long-term use of NSAIDs could result in serious side effects involving the gastrointestinal tract and cardiovascular system [3, 8].

Chondrocytes are the only cells that constitute the cartilage and control the synthesis of cartilage matrix [9]. It is believed that OA results from an imbalance between the synthesis and degradation of ECM [10–12]. Inflammatory cytokines and catabolic factors hinder the functions of chondrocytes and promote the development of OA [13, 14]. Among the inflammatory cytokines, interleukin-1β (IL-1β) is known to initiate the pathogenesis of OA by directly activating nuclear factor kappa-B (NF-κB) and mitogen-activated protein (MAP) kinase, upregulating the expression of cartilage matrix-degrading enzymes such as cyclooxygenase 2 (COX2), inducible nitric oxide synthase (iNOS), and matrix metalloproteinases (MMPs) [15]. Moreover, IL-1β reduces the expression of several anti-inflammatory genes, such as tissue inhibitor of metalloproteinase 3 (TIMP3) and IL-10 [16, 17], inhibits the synthesis of aggrecan and collagen type II (Col2) [18, 19]. These molecules are common indicators of OA. Therefore, we selected iNOS, COX2, MMPs, TIMP3, and IL-10 to evaluate the degradation of ECM in OA.

Members of the dual specificity phosphatase (DUSP) subfamily can directly dephosphorylate the members of the MAPK superfamily (ERK, JNK, and p38) [20, 21]. Moreover, several members of the DUSP subfamily have anti-inflammatory functions. For instance, Yao et al. reported that DUSP19 inhibited IL-1β-induced apoptosis and expression of MMPs in rat chondrocytes via the JAK2/STAT3 signaling pathway [22]. Similarly, Peng et al. showed that DUSP1 inhibited the expression of IL-1β-induced COX-2 and MMPs in rat synoviocytes via the MAPK signaling pathway [23]. Seo et al. reported the anti-inflammatory activity of DUSP5 in lipopolysaccharide (LPS)-stimulated RAW 264.7 cells [24]. In the present study, we investigated the function of DUSP5 in chondrocytes in OA.

## RESULTS

### Human OA knee articular cartilage has reduced DUSP5 expression

**Figure 1:**
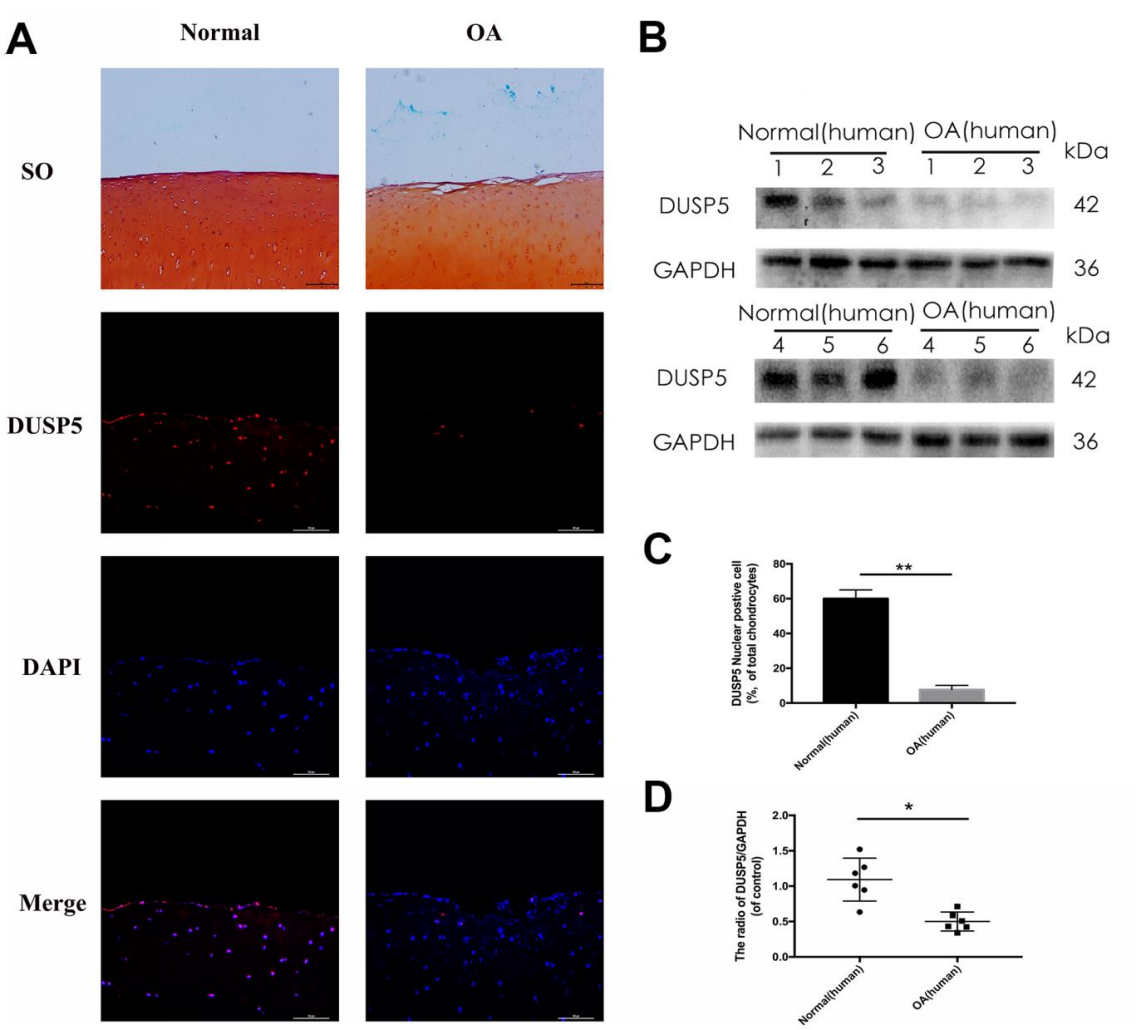
**DUSP5 levels in normal and OA human knee articular cartilage.** (**A**) Representative images of safranin O staining and immunofluorescence staining of DUSP5 in human knee articular cartilage from normal individuals and patients with OA. (**B**, **D**) The protein expression and quantitation of DUSP5 in human chondrocytes derived from normal individuals and patients with OA. GAPDH was used as a control. (**C**) Quantitation of immunofluorescence staining of DUSP5 in human knee articular cartilage from normal individuals and patients with OA. All data are expressed as mean±S.D. (*n* = 3). *p<0.05, **p<0.01.

To ascertain the function of DUSP5 in OA, we first assessed its expression in normal and OA human knee articular cartilage using Safranin O staining and immunofluorescence. Compared with normal tissue, reduced safranin O staining and severe cartilage destruction were observed in OA human knee articular cartilage. Moreover, the expression of DUSP5 decreased in OA human knee articular cartilage (Figure 1A, 1C). Western blotting showed reduced levels of DUSP5 in OA articular cartilage (Figure 1B, 1D). Based on these results, we conclude that the expression of DUSP5 decreased in chondrocytes in OA, indicating that DUSP5 might be inhibited in OA.

### Long-term IL-1β stimulation might inhibit DUSP5 expression

**Figure 2:**
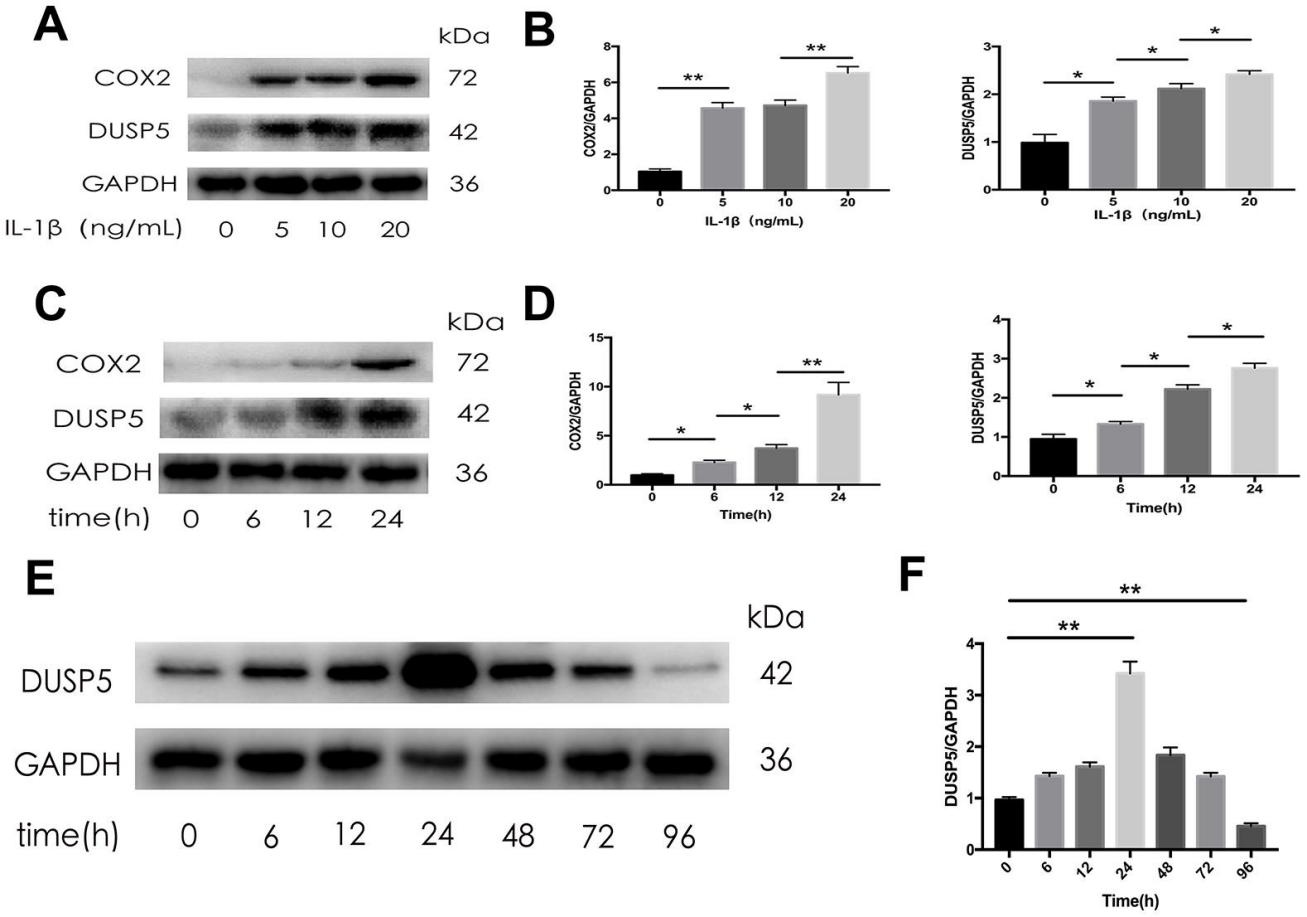
**The expression of DUSP5 in SD rat chondrocytes.** (**A**, **B**) The protein expression and quantitation of DUSP5 in SD rat chondrocytes incubated with IL-1β at 0, 5, 10, and 20 ng/mL for 24 h. (**C**, **D**) The protein expression and quantitation of DUSP5 in SD rat chondrocytes incubated with IL-1β at 10 ng/mL for 0, 6, 12, and 24 h. (**E**, **F**) The protein expression and quantitation of DUSP5 in SD rat chondrocytes incubated with IL-1β at 10 ng/mL for 0, 6, 12, 24, 48, 72, and 96 h. The medium and IL- 1β were added at time Zero. Then every 24 hours changed the medium and re-added IL- 1β. GAPDH was used as the control. All data are expressed as mean±S.D. (*n* = 3). *p<0.05, **p<0.01.

As an *in vitro* OA model, IL-1β has been used to induce inflammation in rat chondrocytes. To investigate the relationship between IL-1β and DUSP5, we detected the expression of DUSP5 after treating the cells with different concentrations of IL-1β for varying time intervals. As shown in Figure 2A–2D, western blotting revealed that IL-1β induced the expression of COX2 and DUSP5 in a time- and concentration-dependent manner. However, the expression of DUSP5 decreased if the IL-1β treatment lasted for more than 24 h (Figure 2E). At 96 h, the expression of DUSP5 was lower than that at 0 h (Figure 2F). Based on these results, we speculate that long-term IL-1β stimulation might inhibit DUSP5 expression.

### Knockdown of DUSP5 aggravates IL-1β-induced inflammation in rat chondrocytes

**Figure 3:**
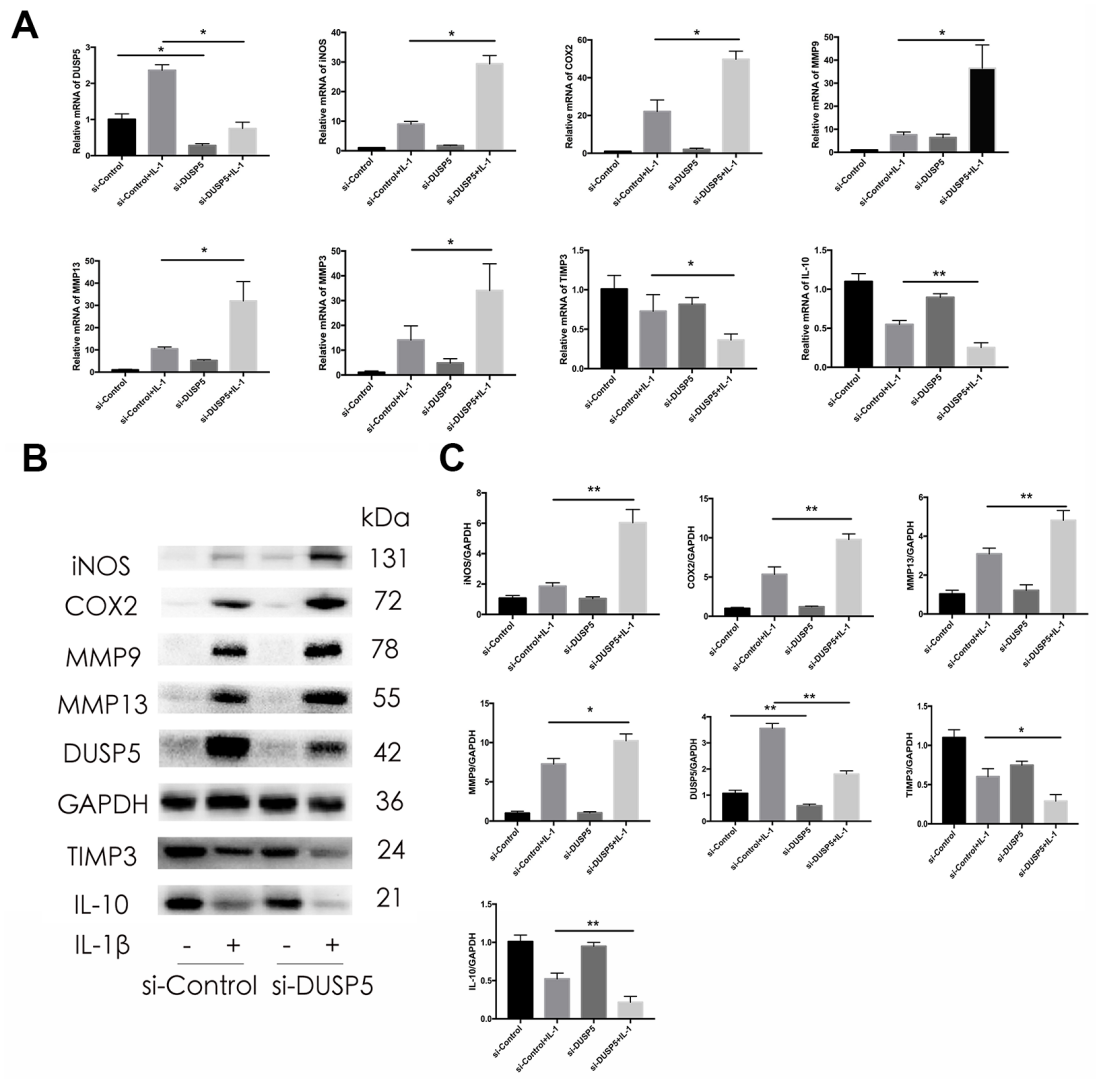
**Effect of DUSP5 knockdown on IL-1β-induced gene expression.** (**A**) Levels of DUSP5, iNOS, COX2, MMP9, MMP13, and MMP3 mRNAs as measured by reverse transcription-quantitative polymerase chain reaction. (**B**, **C**) The protein expression and quantitation of iNOS, COX2, MMP9, MMP13, DUSP5, TIMP3, and IL-10. GAPDH was used as the control. DUSP5 knockdown chondrocytes were incubated with IL-1β (10 ng/mL) for 24 h. All data are expressed as mean±S.D. (*n* = 3). *p<0.05, **p<0.01.

To assess the function of DUSP5 in rat chondrocytes, we transfected the cells with siRNA against DUSP5 (si-DUSP5) or control gene (si-Control). After transfection, chondrocytes were incubated with IL-1β (10 ng/mL) for 24 h. We first detected the transfection efficiency of siRNA. As shown in Figure 3A, 3B, compared with cells transfected with si-Control, the DUSP5 mRNA and protein levels decreased in si-DUSP5-transfected cells, as revealed by reverse transcriptase-polymerase chain reaction (RT-PCR) and western blot analyses. As shown in Figure 3A, compared with cells transfected with si-Control + IL-1β, those transfected with si-DUSP5 + IL-1β had elevated mRNA levels of cartilage matrix-degrading enzymes such as iNOS, COX2, MMP9, MMP13, and MMP3. Further, compared with cells treated with si-Control + IL-1β, those treated with si-DUSP5 + IL-1β had reduced mRNA levels of anti-inflammatory genes, TIMP3 and IL-10 (Figure 3B). Western blotting showed increased expression of iNOS, COX2, MMP9, and MMP13, and reduced expression of TIMP3 and IL-10 in si-DUSP5 + IL-1β-treated chondrocytes as compared with the levels in si-Control + IL-1β-treated cells (Figure 3B, 3C). These results showed that the knockdown of DUSP5 aggravated IL-1β-induced inflammation.

### Overexpression of DUSP5 ameliorates IL-1β-induced inflammation in rat chondrocytes

**Figure 4:**
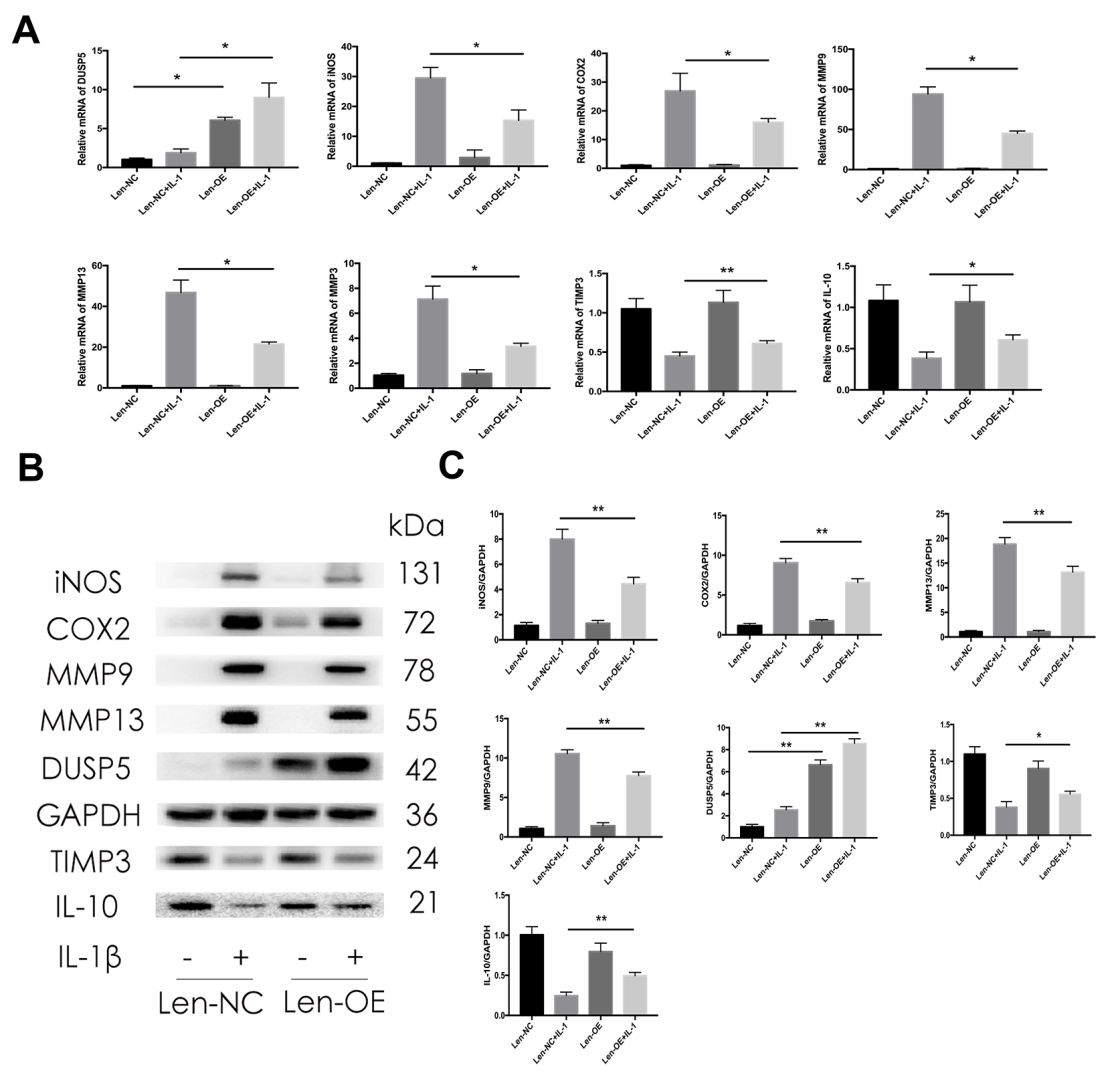
**Effect of DUSP5 overexpression on IL-1β-induced gene expression.** (**A**) Levels of DUSP5, iNOS, COX2, MMP9, MMP13, and MMP3 mRNAs measured by reverse transcription-quantitative polymerase chain reaction. (**B**, **C**) The protein expression and quantitation of iNOS, COX2, MMP9, MMP13, DUSP5, TIMP3, and IL-10. GAPDH was used as the control. DUSP5 overexpressed chondrocytes were incubated with IL-1β (10 ng/mL) for 24 h. All data are expressed as mean±S.D. (*n* = 3). *p<0.05, **p<0.01.

To confirm the protective effect of DUSP5 on IL-1β-induced chondrocytes, we used lentivirus particles overexpressing DUSP5 (Len-OE) to overexpress DUSP5. Lentivirus-control particles (Len-NC) served as the control. We first detected the transduction efficiency of the lentivirus system. As shown in Figure 4A, 4B, compared with cells transduced with Len-NC, those transduced with Len-OE showed increased mRNA and protein levels of DUSP5, as revealed by RT-PCR and western blot analyses, respectively. As shown in Figure 4A, the stimulatory effects of IL-1β on iNOS, COX2, MMP9, MMP13, and MMP3 decreased in Len-OE + IL-1β-treated chondrocytes at mRNA levels than in cells treated with Len-NC + IL-1β. Further, the mRNA levels of anti-inflammatory genes, TIMP3 and IL-10 increased in Len-OE + IL-1β-treated chondrocytes as compared with those in Len-NC + IL-1β-treated cells (Figure 4A). Western blotting revealed that compared with Len-NC + IL-1β-treated chondrocytes, the expression of iNOS, COX2, MMP9, and MMP13 decreased and that of TIMP3 and IL-10 increased in Len-OE + IL-1β-treated chondrocytes (Figure 4B, 4C). These findings demonstrated that the overexpression of DUSP5 ameliorated IL-1β-induced inflammation in rat chondrocytes.

### DUSP5 restrains IL-1β-induced inflammation and protects chondrocytes by suppressing the ERK and NF-κB signaling pathways

**Figure 5:**
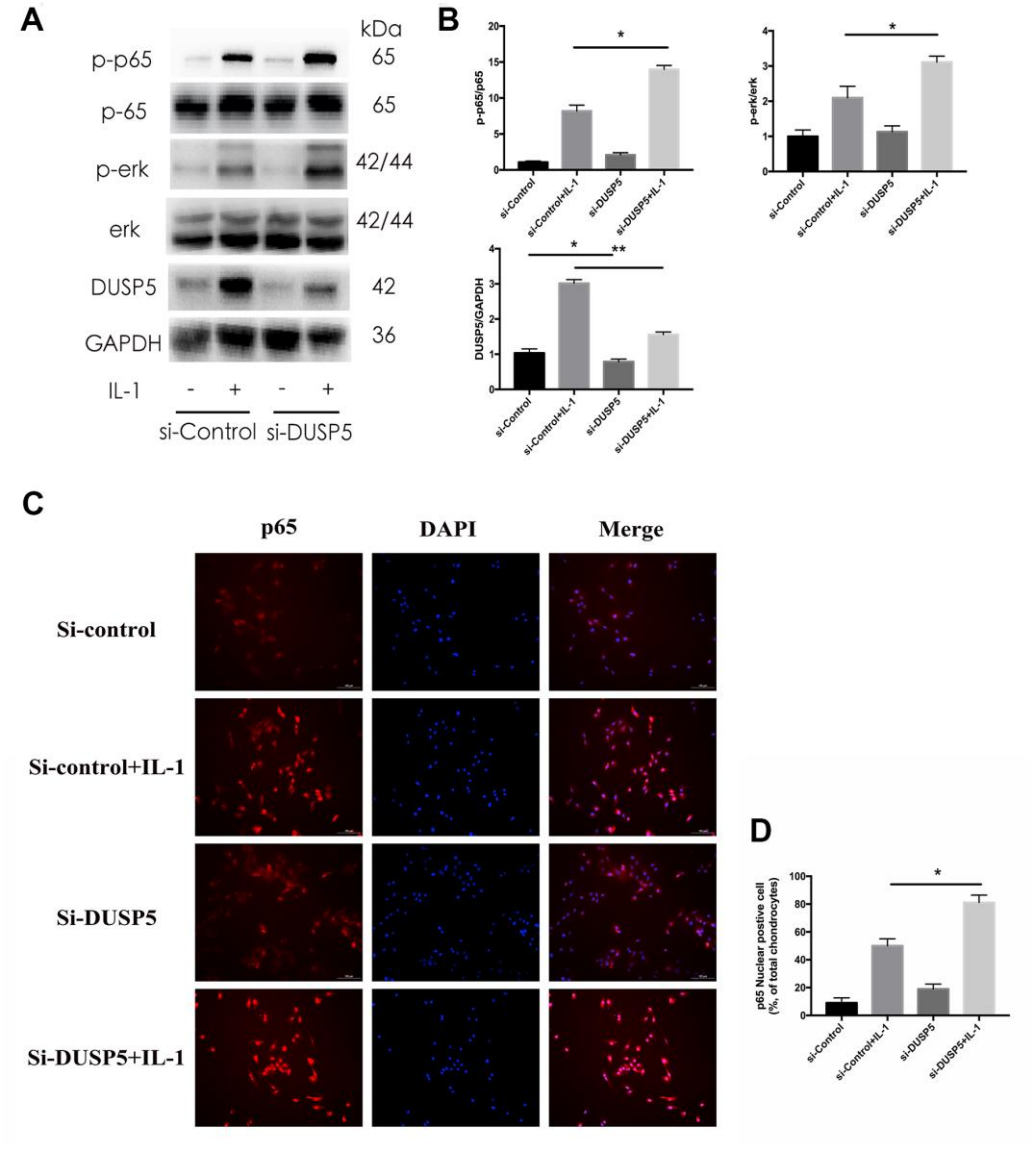
**Effect of DUSP5 knockdown on IL-1β-induced NF-κB and ERK signaling pathways in chondrocytes.** (**A**, **B**) The protein expression and quantitation of DUSP5, p-p65, and p-ERK. GAPDH was used as the control. (**C**) The nuclear translocation of p65 was detected by immunofluorescence; DAPI was used to stain the DNA. Blue, DAPI; red, p65. (**D**) Quantitation of immunofluorescence staining of p65. DUSP5 knockdown chondrocytes were incubated with IL-1β (10 ng/mL) for 10 min. All data are expressed as mean±S.D. (*n* = 3). *p<0.05, **p<0.01.

**Figure 6:**
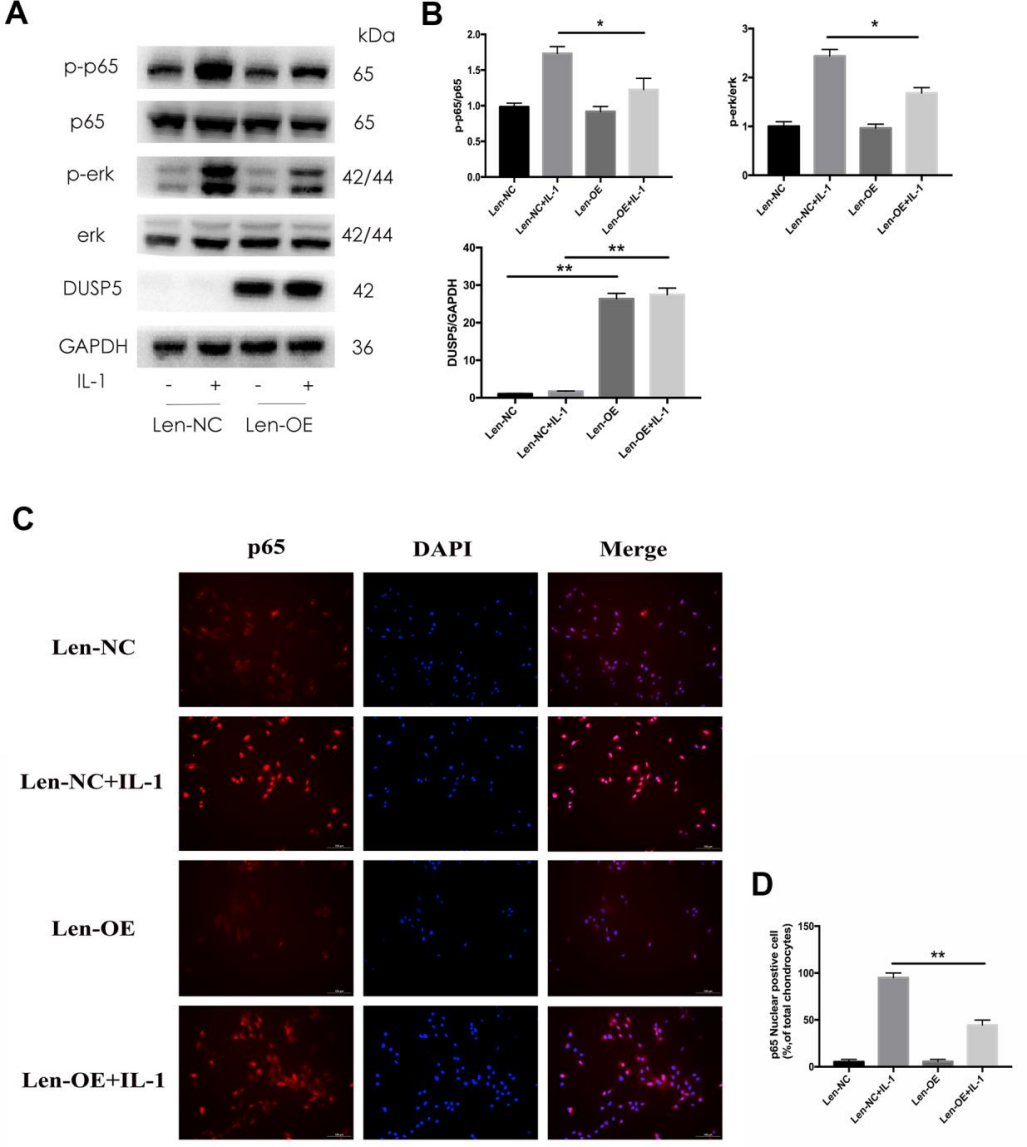
**Effect of DUSP5 overexpression on IL-1β-induced NF-κB and ERK signaling pathways in chondrocytes.** (**A**, **B**) The protein expression and quantitation of DUSP5, p-p65, and p-ERK. GAPDH was used as the control. (**C**) The nuclear translocation of p65 was detected by immunofluorescence; DAPI was used to stain the DNA. Blue, DAPI; red, p65. (**D**) Quantitation of immunofluorescence staining of p65. DUSP5 overexpressed chondrocytes were incubated with IL-1β (10 ng/mL) for 10 min. All data are expressed as mean±S.D. (*n* = 3). *p<0.05, **p<0.01.

Multiple signaling pathways are known to function in the development of OA. Seo et al. [24] reported that DUSP5 suppressed the NF-κB and ERK pathways in LPS-stimulated RAW 264.7 cells. Therefore, we next investigated the status of these pathways. Rat chondrocytes were transfected with si-DUSP5, followed by incubation with IL-1β (10 ng/mL) for 10 min. As shown in Figure 5A, 5B, the expression of p-ERK and p-p65 increased in si-DUSP5 + IL-1β-treated cells as compared with that in si-Control + IL-1β-treated cells. In addition, immunofluorescence revealed that IL-1β induced greater translocation of NF-κB to the nucleus in si-DUSP5 + IL-1β-treated cells than in si-Control+ IL-1β-treated cells (Figure 5C, 5D). Further, western blotting revealed that compared with Len-NC + IL-1β-treated cells, the expression of p-ERK and p-p65 decreased in Len-OE + IL-1β-treated cells (Figure 6A, 6B). Immunofluorescence showed that IL-1β induced less translocation of NF-κB to the nucleus in Len-OE + IL-1β-treated cells than in Len-NC + IL-1β-treated cells (Figure 6C, 6D). Altogether, these results showed that DUSP5 suppressed the IL-1β-induced inflammation by inhibiting the NF-κB and ERK signaling pathways.

### Inhibition of NF-κB and ERK signaling pathways ameliorates IL-1β-induced inflammation in si-DUSP5 chondrocytes

**Figure 7:**
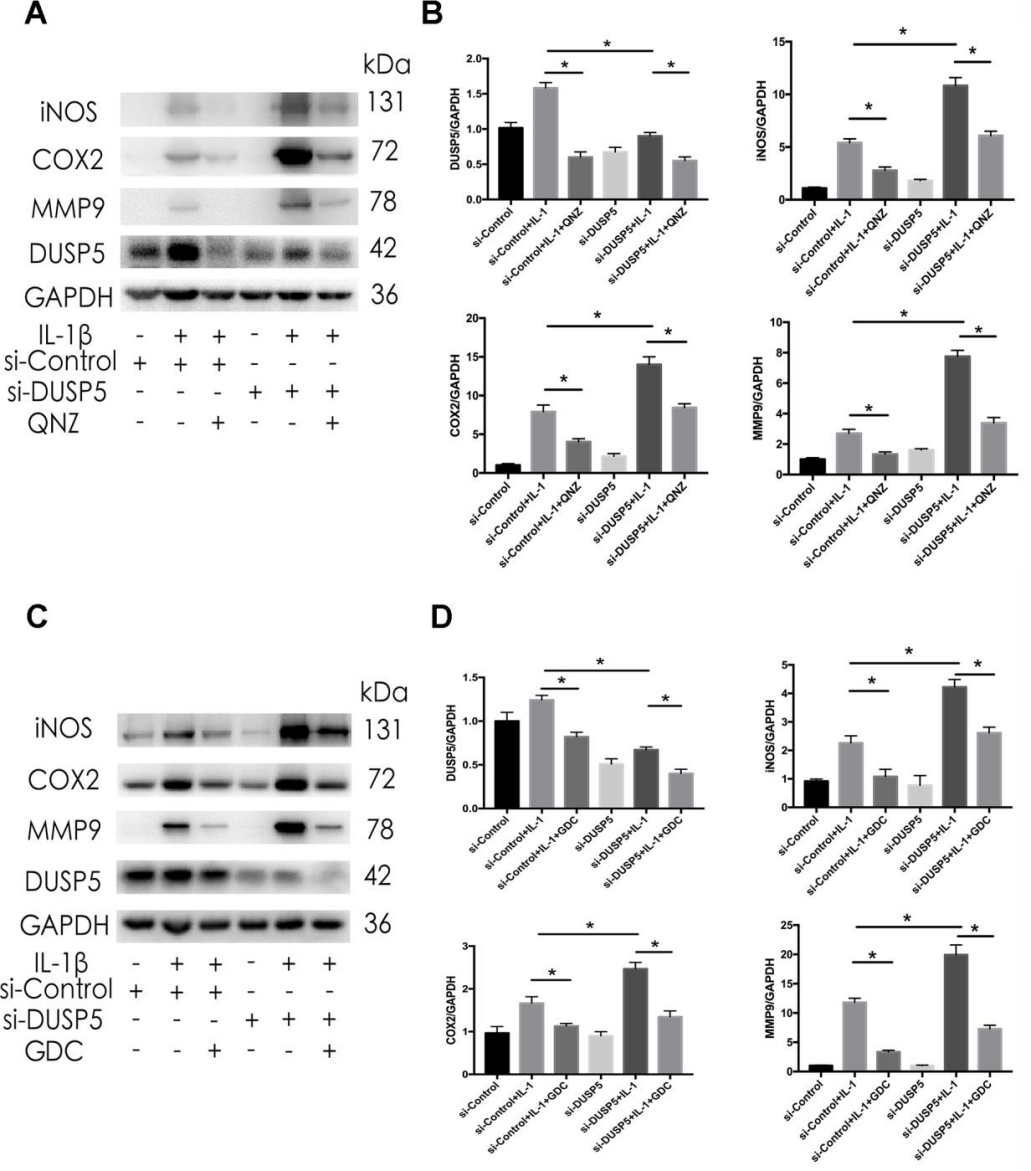
**Effect of QNZ and GDC on IL-1β-induced inflammation in DUSP5 knockdown chondrocytes.** (**A**, **B**) The protein expression and quantitation of DUSP5, iNOS, COX2, and MMP9. (**C**, **D**) The protein expression and quantitation of DUSP5, iNOS, COX2, and MMP9. GAPDH was used as the control. DUSP5 knockdown chondrocytes were treated with 10 μM QNZ or 6.1 nM GDC for 2 h before incubation with IL-1β (10 ng/mL) for 24 h. All data are expressed as mean±S.D. (*n* = 3). *p<0.05, **p<0.01.

To further study the involvement of NF-κB and ERK pathways in the pathogenesis of OA, we inhibited these pathways in cells transfected with si-DUSP5. The si-DUSP5-transfected chondrocytes were treated with either 10 μM QNZ (inhibitor of NF-κB) [25] or 6.1 nM GDC (inhibitor of ERK) [26] for 2 h, followed by treatment with IL-1β. First, we assessed the effectiveness of QNZ and GDC. As shown in Supplementary Figure 1A, 1B, QNZ and GDC could effectively inhibit the expression of p-p65 and p-ERK, respectively. As shown in Figure 7A–7D, western blotting revealed that IL-1β-induced expression of iNOS, COX2, and MMP9 decreased following the treatment with QNZ or GDC. These results showed that DUSP5 restrained the IL-1β-induced inflammation by suppressing the NF-κB and ERK signaling pathways.

### DUSP5 ameliorates osteoarthritis in rat OA model

**Figure 8:**
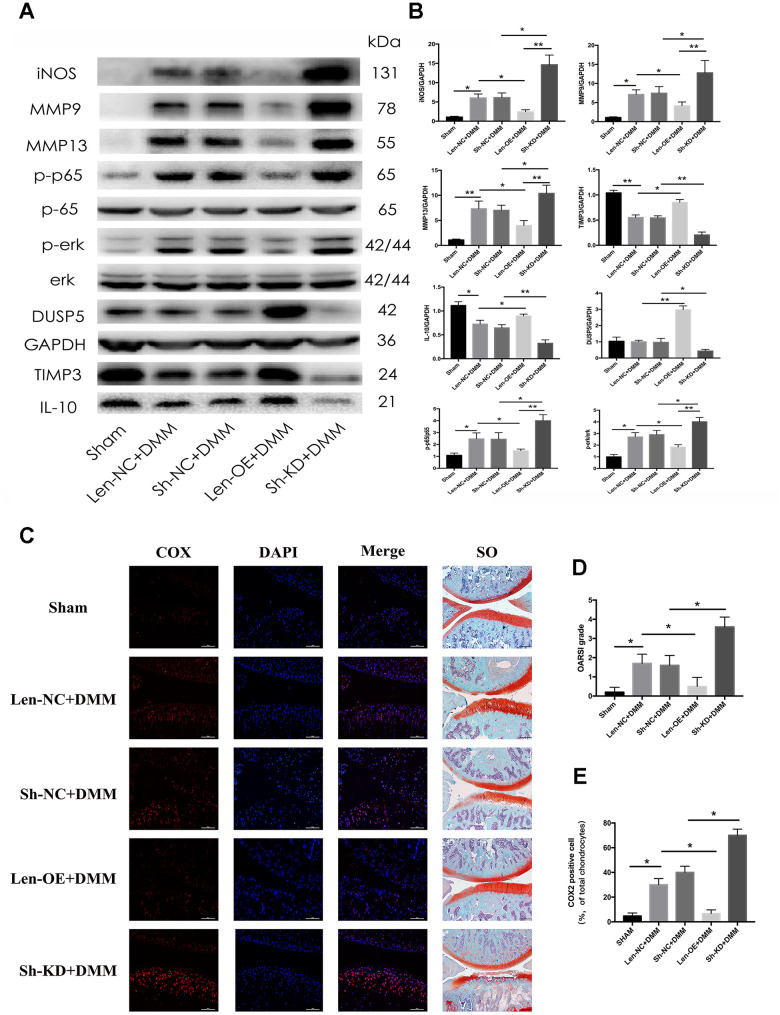
**Effect of DUSP5 overexpression and knockdown on rat OA model.** Fifty rats were randomly divided into five groups (10 rats in each group). The rats in the Sham group received sham surgery as blank control. The other 40 rats underwent DMM surgical removal of the medial meniscus and were regarded as OA rats. Lentivirus particles overexpressing DUSP5 (1×10^8^ TU/mL) were injected intra-articularly into rats in the Len-OE group. Lentivirus-shDUSP5 particles (1×10^8^ TU/mL) were injected intra-articularly into rats in the Sh-KD group. Lentivirus-control particles (1×10^8^ TU/mL) were injected intra-articularly into rats in the Len-NC group used as the control for Len-OE. Lentivirus-shControl particles (1×10^8^ TU/mL) were injected intra-articularly into rats in the Sh-NC group used as the control for Sh-KD. PBS was injected intra-articularly into rats in the Sham group. Rats were sacrificed at 8 weeks post-DMM surgery from each group. (**A**, **B**) The protein expression and quantitation of iNOS, MMP9, MMP13, p-p65, p-ERK, DUSP5, TIMP3, and IL-10. GAPDH was used as the control. (**C**) Microscopic images of COX2 immunofluorescence analysis and safranin O staining of rat knee joint sections. Blue, DAPI; red, COX2. (**D**) The OARSI grading system (0–6) (obtained from experiments of 10 knees each group) was used to evaluate the sections. (**E**) Quantitation of immunofluorescence staining of COX2. *p < 0.05, **p<0.01.

Next, we studied the function of DUSP5 *in vivo*. We performed destabilization of the medial meniscus (DMM) surgery in rats to establish an animal model for OA. Len-OE and lentivirus-shDUSP5 particles (Sh-KD) were injected intra-articularly into OA rats to overexpress or knockdown DUSP5, respectively. The Len-NC and lentivirus-shControl (Sh-NC) particles served as controls for overexpression and knockdown experiments. RT-PCR and western blotting confirmed the efficiency of Sh-KD or Len-OE *in vivo*. As shown in Supplementary Figure 1C and Figure 8A, 8B, compared with each control group, the expression of DUSP5 reduced in Sh-KD-transduced rats and increased in Len-OE-transduced rats. Furthermore, we detected the inflammatory markers, anti-inflammatory markers, NF-κB and ERK pathways. As shown in Figure 8A, 8B, compared with Len-NC + DMM-treated rats, the expression of iNOS, MMP9, MMP13, NF-κB, and ERK decreased, TIMP3 and IL-10 increased in Len-OE + DMM-treated rats. Compared with Sh-NC + DMM-treated rats, the expression of iNOS, MMP9, MMP13, NF-κB, and ERK increased, TIMP3 and IL-10 decreased in Sh-KD + DMM-treated rats (Figure 8A, 8B). Further, immunofluorescence results showed higher expression of COX2 in Sh-KD + DMM-treated rats than in Sh-NC + DMM-treated rats and less expression of COX2 in Len-OE + DMM-treated rats than in Len-NC + DMM-treated rats (Figure 8C, 8E). The safranin O staining results showed that Len-OE + DMM-treated rats had considerably less cartilage destruction than Len-NC + DMM-treated rats, whereas Sh-KD + DMM-treated rats had more severe cartilage destruction than Sh-NC + DMM-treated rats. As compared with each control group, the OARSI score decreased in Len-OE + DMM-treated rats and increased in Sh-KD + DMM-treated rats (Figure 8D) [27]. Altogether, these findings demonstrated that DUSP5 restrained IL-1β-induced inflammation and ameliorated OA in rats by suppressing the NF-κB and ERK signaling pathways.

## DISCUSSION

Dual specificity phosphatase 5 (DUSP5) is a member of the dual specificity phosphatase (DUSP) subfamily, and can be induced by several cytokines in mammalian cells [28, 29]. Recent studies reported that DUSP5 specifically inactivated ERK in the nucleus and no other MAP kinases [30, 31]. DUSP5 has a highly conserved kinase interaction motif (KIM) that can specifically recognize and bind to ERK to inactivate it [32]. Moreover, increased expression of DUSP5 not only dephosphorylated ERK but also translocated ERK to the nucleus via a nuclear localization signal (NLS) [33].

Although DUSP5 is expressed in various cell types, not many studies have been conducted to ascertain its functions. Some studies have revealed that DUSP5 has an anti-inflammatory function and participates in the pathogenesis of several diseases, including pulmonary fibrosis, autoimmune arthritis, cardiovascular disease, and cancer [31, 34, 35]. Moon et al. reported that the overexpression of DUSP5 reduced the expression IgG, IgG1, and IgG2a in a rheumatoid arthritis (RA) mouse model circulatory system by downregulating the activity of p-ERK and inhibiting the expression of tumor necrosis factor alpha (TNF-α) and IL-1β [34]. Habibian et al. showed that DUSP5 inhibited the expression of COX2, IL-16, and ERK in adipocytes of a high-fat diet mouse model [36]. However, Jo et al. reported that although the overexpression of DUSP5 inhibited the expression of ERK, it increased the endoplasmic reticulum (ER) stress response in hepatocytes, resulting in hepatocytes death via the PERK–CHOP pathway [37]. In this study, we found reduced expression of DUSP5 in human OA knee articular cartilage, revealing a protective role of DUSP5 in ameliorating OA.

Osteoarthritis (OA) is considered a disease of the whole joint, characterized by cartilage degeneration, synovial inflammation, and subchondral sclerosis [38]. The imbalance between the expression of matrix-degrading and anti-inflammatory genes triggers the degeneration of ECM [39]. It has been demonstrated that MMPs, whose levels are increased in OA, accelerate the cartilage degeneration, whereas TIMPs decrease the cartilage degradation by inhibiting MMPs [40]. Moreover, it was demonstrated that IL-10 reduced cartilage degradation by decreasing the expression of MMPs and pro-inflammatory cytokines [41]. Therefore, inhibiting the expression of matrix-degrading genes and reducing chondrocyte inflammation could be a potential therapeutic strategy for OA [42, 43].

In this study, we revealed that overexpressed DUSP5 reduced the IL-1β-induced expression of inflammatory genes, including iNOS, COX2, MMP9, and MMP13. Moreover, IL-1β-induced downregulation of TIMP3 and IL-10 was restored by overexpressed DUSP5. These results demonstrate that DUSP5 has an anti-inflammatory function in chondrocytes *in vitro*. In addition, *in vivo* results confirmed the protective role of DUSP5 in ameliorating cartilage erosion and reducing matrix degeneration.

Next, we investigated the potential mechanism behind the anti-inflammatory effect of DUSP5. The continuous activation of the NF-κB pathway has been reported in several cancers and chronic inflammatory diseases [44]. Yasuda et al. reported that chondrocytes have multiple cytokine receptors (such as IL-1β receptor), which get activated after binding to their corresponding ligands (such as IL-1β) and induce the NF-κB signaling pathway and the expression of several inflammatory genes such as MMPs, iNOS, and COX2 [45].

The MAPK signaling pathway is known to be involved in chondrocyte proliferation, apoptosis, and MMPs regulation [46, 47]. Studies have demonstrated high levels of phosphorylated ERK, JNK, and p38 in the cartilage tissue of patients with OA [48, 49]. Both *in vitro* and *in vivo* studies have proved that inhibitors of ERK, JNK, and p38 suppressed the chondrocyte inflammation [50, 51].

This study, we revealed NF-κB and ERK could be activated by IL-1β, then induce inflammation in rat chondrocytes. Inhibitor of NF-κB or ERK could inhibit the activation of NF-κB or ERK and suppress the IL-1β induced inflammation. Our data demonstrated that DUSP5 exerts its anti-inflammatory effect by inhibiting the activation of NF-κB and ERK signaling pathways.

Interestingly, we found reduced expression of DUSP5 in the chondrocytes of patients with OA. The expression of DUSP5 increased in a time-dependent manner with IL-1β treatment; however, it declined when the IL-1β treatment lasted for more than 24 h. Similarly, Habibian et al. reported that TNF-α transiently (<1 h) increased the DUSP5 mRNA expression, with levels returning to normal in <2 h in mature adipocytes [36]. Chen et al. reported that DUSP5 is unstable in the nucleus due to its short half-life and is easily degraded by the ubiquitin-proteasome system (UPS) [32, 33]. It is not clear whether the reason for the decrease of DUSP5 expression is its own instability or the inhibition of long-term IL-1β. Further studies are required to understand the decrease of DUSP5.

Although our study provided promising results, it had a few limitations. The chondrocytes used in our research were derived from rats, which may not completely mimic the characteristics of human chondrocytes. Studies in human chondrocytes are warranted to prove the anti-inflammatory effect of DUSP5 further. Besides, OA is a complex disease and many types of cells participate in the pathogenesis of OA, such as chondrocytes, synovial cells and osteoclasts [2]. However, function of DUSP5 in synovial cells and osteoclasts is not clear. Besides, it is only known that DUSP5 can directly dephosphorylate ERK, but it is not clear how DUSP5 regulates NF-κB. In addition, the upstream mechanism of DUSP5 is unclear and this is our next research direction.

We believe the future of modern medicine is gene therapy. Gene therapy is, potentially, a powerful tool for treating diseases such as amyotrophic lateral sclerosis (ALS), Parkinson's disease (PD), Alzheimer's disease (AD) and hemophilia. ONPATTRO™ (Patisiran), the first RNA interference (RNAi) therapeutic drug has been approved by FDA for the treatment of the polyneuropathy of hereditary transthyretin-mediated (hATTR) amyloidosis in adults [52]. Besides, clinical trials have proved adeno-associated virus (AAV)-mediated gene therapy for patients with hemophilia A is effective and safe [53]. Our vision is to find the key genes in osteoarthritis, then use adeno-associated virus (AAV)-mediated gene therapy or RNA interference (RNAi) therapy to delay or even reverse osteoarthritis. A lot of research is needed in the future.

In conclusion, we demonstrated reduced expression of DUSP5 in human OA cartilage. Our *in vitro* and *in vivo* experiments showed that DUSP5 decreased the IL-1β-induced inflammation in rat chondrocytes by inhibiting the NF-κB and ERK signaling pathways and ameliorated OA. These results present DUSP5 as a potential therapeutic target for OA.

## MATERIALS AND METHODS

### Reagents

Recombinant IL-1β was purchased from R&D Systems, UK. Trypsin (0.25%), fetal bovine serum (FBS), Dulbecco’s modified Eagle’s medium (DMEM), and penicillin/streptomycin were obtained from Gibco RRL, USA. Bovine serum albumin (BSA), type II collagenase, and safranin O dye were purchased from Sigma-Aldrich, USA. Phosphatase and protease inhibitors, and TRIzol reagent were purchased from Invitrogen, Grand Island, NY, USA. QNZ (EVP4593) and ravoxertinib (GDC-0994) were purchased from Selleckchem, Houston, TX, USA.

### Human tissue samples

This study was approved by the Ethics Committee of The Second Affiliated Hospital, School of Medicine, Zhejiang University, Hangzhou, China. Human OA cartilage samples were obtained from six patients (4 females [age: 55, 58, 74, 77 years] and 2 males [age: 62, 67 years]) undergoing total knee joint replacement surgery at The Second Affiliated Hospital. Normal human articular cartilage samples were obtained from six patients (3 females [age: 57, 63, 69 years] and 3 males [mean age: 54, 63, 70 years]) during the amputation surgery at The Second Affiliated Hospital. All patients undergoing amputation surgery were amputated due to a thigh injury caused by traffic accidents. No obvious damage to knee cartilage was reported.

### Cell culture

All animal studies were conducted in accordance with the Declaration of Helsinki, and the protocol was approved by the Ethics Committee of the Second Affiliated Hospital, School of Medicine, Zhejiang University, Hangzhou, China. Articular cartilage was obtained from 3-week-old Sprague–Dawley (SD) rats (Zhejiang Academy of Medical Sciences, Hangzhou, China) euthanized with an overdose of pentobarbital sodium. Next, the articular cartilages were minced into 1 to 3 mm-thick pieces and digested with 0.2% type II collagenase at 37° C for 4 h. Next, the chondrocytes were suspended and seeded into 25 cm^2^ flasks with DMEM/F12 (10% FBS, 1% penicillin/streptomycin) at 37° C in an atmosphere containing 5% CO_2_. These cells were considered passage 0 (P0). After reaching confluency of approximately 90%, chondrocytes were trypsinized using 0.25% Trypsin-EDTA and passaged at a ratio of 1:3.

### Knockdown experiments using small interfering RNA

The following siRNA sequences were used for performing transfections:

si-DUSP5 (Sense: 5’-GCACAACCCACCUACACUATT-3’; Anti-sense: 5’-UAGUGUAGGUGGGUUGUGCTT-3’) and si-Control (Sense: 5’-UUCUCCGAACGUGUCACGUTT-3’; Anti-sense: 5’-ACGUGACACGUUCGGAGAATT-3’).

The oligonucleotide sequences were obtained from Sangon Biotech (Shanghai, China). For transfections, chondrocytes (confluency ~30–50%) were incubated with Lipofectamine 3000 transfection reagent (Thermo Fisher, USA) in the growth medium for 4 to 6 h according to the manufacturer’s protocol. Next, the medium was replaced with fresh growth medium. The chondrocytes were used for further experiments.

### Overexpression experiments using lentiviral particles

Lentivirus particles overexpressing DUSP5 (Len-OE) and lentivirus-control particles (Len-NC) were obtained from GeneChem, Shanghai, China. For transductions, chondrocytes (confluency ~30–50%) were incubated with lentiviral particles in the growth medium (growth medium:lentiviral particles =1 mL:10 μL) for 24 h according to the manufacturer’s protocol. After 24 h, the medium was replaced with fresh growth medium. Next, the chondrocytes were cultured for 2 days for further experiments.

### Real-time polymerase chain reaction

Chondrocytes (1 × 10^5^) were seeded in six-well plates. After treatment with 10 ng/mL IL-1β for 24 h, cells were harvested for quantitative RT-PCR. The total RNA was extracted using the TRIzol reagent (Invitrogen). The concentrations of RNA were detected via a nucleic acid detector. The RNA was reverse-transcribed to cDNA using the PrimeScript 1^st^ Strand cDNA Synthesis Kit (TaKaRa; Dalian, China). GAPDH was used as the endogenous control, and the expression of iNOS, COX2, MMP3, MMP9, MMP13, and DUSP5 was analyzed by RT-PCR (Bio-Rad; Marnes-la-Coquette, France). All primers used are shown in Table 1. Relative gene expression was calculated using the 2^(-ΔΔCt)^ method. All experiments were performed in triplicate.

**Table 1:** Primer sequences used in RT-PCR in the study.

**Gene**	**Forward**	**Reverse**
GAPDH	5’- GAAGGTCGGTGTGAACGGATTTG -3’	5’-CATGTAGACCATGTAGTTGAGGTCA-3’
DUSP5	5’-CTCACCTCGCTGCTGGCCTGTCTG-3’	5’-GCCTCTTTCACCTTCGAGCTTCTC-3’
iNOS	5’-GATAACCATCCGAGCGACCTTT-3’	5’-CAGTTTGAGAGAGGAGGCTCCG-3’
COX2	5’-GAGAGATGTATCCTCCCACAGTCA-3’	5’-GACCAGGCACCAGACCAAAG-3’
MMP3	5’-CAGGCATTGGCACAAAGGTG-3’	5’-GTGGGTCACTTTCCCTGCAT-3’
MMP9	5’-AGCCGGGAACGTATCTGGAA-3’	5’-CCGGTTGTGGAAACTCACAC-3’
MMP13	5’-GCAAACCCTGCGTATTTCCAT-3’	5’-GATAACCATCCGAGCGACCTTT-3’
TIMP3	5’-CCAGAAAGAATGAGACCGCAC-3’	5’-TGGGGTGTACATACGGGAGA-3’
IL-10	5’-ATAAAAGCAAGGCAGTGGAGC-3’	5’-GCCGGGTGGTTCAATTTTTC-3’

### Western blotting

After the cells were treated with IL-1β, total protein was extracted from chondrocytes by lysing the cells with radioimmunoprecipitation assay (RIPA) buffer containing protease and phosphatase inhibitors for 40 min. Next, the bicinchoninic acid (BCA) protein assay kit (Beyotime Biotechnology; Shanghai, China) was used to quantify the samples. The samples were separated on a 10% sodium dodecyl sulfate-polyacrylamide (SDS-PAGE) gel, and subsequently transferred onto a polyvinylidene difluoride (PVDF) membrane. Afterward, the membrane was blocked with 5% BSA for 1 h and cut into different blots based on the molecular weights of proteins to be checked. The blots were incubated overnight at 4° C with corresponding primary antibodies against GAPDH (rabbit mAb; ab70699; Abcam), DUSP5 (rabbit mAb; ab200708; Abcam), MMP9 (rabbit mAb; ab76003; Abcam), MMP13 (rabbit mAb; sc-30073; Santa Cruz Biotechnology, Inc.), COX2 (rabbit mAb; 12282; Cell Signaling Technology, Inc.), iNOS (rabbit mAb; ab3523; Abcam), TIMP3 (rabbit mAb; ab39184; Abcam), IL-10 (rabbit mAb; ab192271; Abcam), p65 (rabbit mAb; 4764S; Cell Signaling Technology, Inc.), p-p65 (rabbit Ab; 3031; Cell Signaling Technology, Inc.), ERK (rabbit mAb; 4695; Cell Signaling Technology, Inc.), and p-ERK (rabbit mAb; 4370; Cell Signaling Technology, Inc.). After washing with TBST (Tris-buffered saline with Tween 20) thrice, the blots were incubated with corresponding secondary antibodies with 5% BSA for 1 h at room temperature. Finally, the protein bands were luminesced using Pierce ECL western blotting substrate and detected with the ChemiDoc Imaging System (Bio-Rad). All assays were performed in triplicate. “Quantity One” software was used to determine the gray value of the bands.

### Animal osteoarthritis model

Fifty male Sprague–Dawley rats (200–250 g; 6-week-old), obtained from Zhejiang Academy of Medical Sciences, Hangzhou, China, were randomly divided into five groups (10 rats/5 cages in each group). We performed destabilization of the medial meniscus (DMM) surgery to establish an OA model. First, rats were anesthetized by pentobarbital sodium (40 mg/kg). Next, the medial parapatellar approach was used to expose the knee, and a medial capsular incision was made. The patella was laterally retracted to expose the medial meniscus. Subsequently, the medial meniscus was removed. The wound was closed in layers by sutures: The incision of medial capsular was first closed by absorbable sutures, and subsequently the skin was closed. Sham surgery was performed in rats in the Sham group using the same surgical approach without removal of the medial meniscus.

Forty rats (four groups) received the DMM surgery (OA rats), and 10 rats (remaining group) received the sham surgery (control). Lentivirus particles overexpressing DUSP5 (1 × 10^8^ TU/mL) were injected intra-articularly into rats in the Len-OE group. Lentivirus-shDUSP5 particles (1 × 10^8^ TU/mL) (sense: 5’-GCACAACCCACCTACACTA-3’, loop: CTCGAG, anti-sense: 5’-TAGTGTAGGTGGGTTGTGC -3’) were injected intra-articularly into rats in the Sh-KD group. Lentivirus-control particles (1 × 10^8^ TU/mL) were injected intra-articularly into rats in the Len-NC group and used as the control for Len-OE. Lentivirus-shControl particles (1 × 10^8^ TU/mL) were injected intra-articularly into rats in the Sh-NC group and used as the control for Sh-KD. Phosphate-buffered saline (PBS) was injected intra-articularly into rats in the Sham group. Intra-articular injection of 20 μL lentivirus particles or PBS was administered to rats in each group at 7 days before the surgery and 7, 21, and 35 days after the surgery. Intra-articular injection was administered as follows: First, rats were anesthetized by pentobarbital sodium (40 mg/kg). Next, an iodophor was used to disinfect the rat knee joint. A micro-syringe was used to aspirate 20 μL lentivirus particles or PBS. On the lateral side of the patella, the knee joint was penetrated along the quadriceps tendon, following which lentivirus particles or PBS was injected. Next, the knee joints of rats were flexed and extended to allow uniform distribution of lentivirus particles or PBS.

Rats from each group were sacrificed at 8 weeks post-DMM surgery. The knee joints were dissected and preserved in 4% paraformaldehyde solution. The study was conducted in accordance with the National Institutes of Health (NIH) guidelines (NIH Pub no. 85-23, revised 1996), and the protocol was approved by the Ethics Committee of the Second Affiliated Hospital, School of Medicine, Zhejiang University, Hangzhou, China.

### Safranin O staining and immunofluorescence

The histological sections (3.5-mm thick) of human and rat cartilages were prepared using a microtome. Next, the sections were stained with safranin O [54] and prepared for immunofluorescence [55] according to the protocols described earlier [52, 53]. The OARSI grading system (0–6) was used to evaluate the sections [27].

### Immunofluorescence

Chondrocytes were seeded in 24-well plates and grown to approximately 50% confluency. Next, the cells were treated with 10 ng/mL IL-1β for 10 min. After washing with PBS thrice, chondrocytes were fixed in 4% paraformaldehyde and permeabilized using 0.3% Triton X-100 for 15 min. After blocking with 5% BSA for 1 h at 37° C, cells were incubated with anti-p-65 antibody overnight at 4° C. The cells were washed and incubated with secondary antibody for 1 h at room temperature and subsequently stained with DAPI for 5 min. The cells were observed using fluorescence microscopy.

### Statistical analysis

All experiments were performed in triplicate using independent samples. All data are expressed as means ± standard deviations (SDs) and were analyzed using a one-way analysis of variance. Statistical analyses were performed using the SPSS software version 19.0. The value of *p* < 0.05 was considered significant.

### Data availability

All data that support the findings of this study are included in the article.

## Supplementary Material

Supplementary Figure 1
