# Supplementary material for: DUSP5 suppresses interleukin-1β-induced chondrocyte inflammation and ameliorates osteoarthritis in rats
Source: Aging (Albany NY). 2020 Dec 15;12(24):26029–46. doi: 10.18632/aging.202252 (PMC7803505; doi:10.18632/aging.202252)
Supplement: Supplementary Figure 1 [file aging-12-202252-s001.pdf]

SUPPLEMENTARY FIGURE

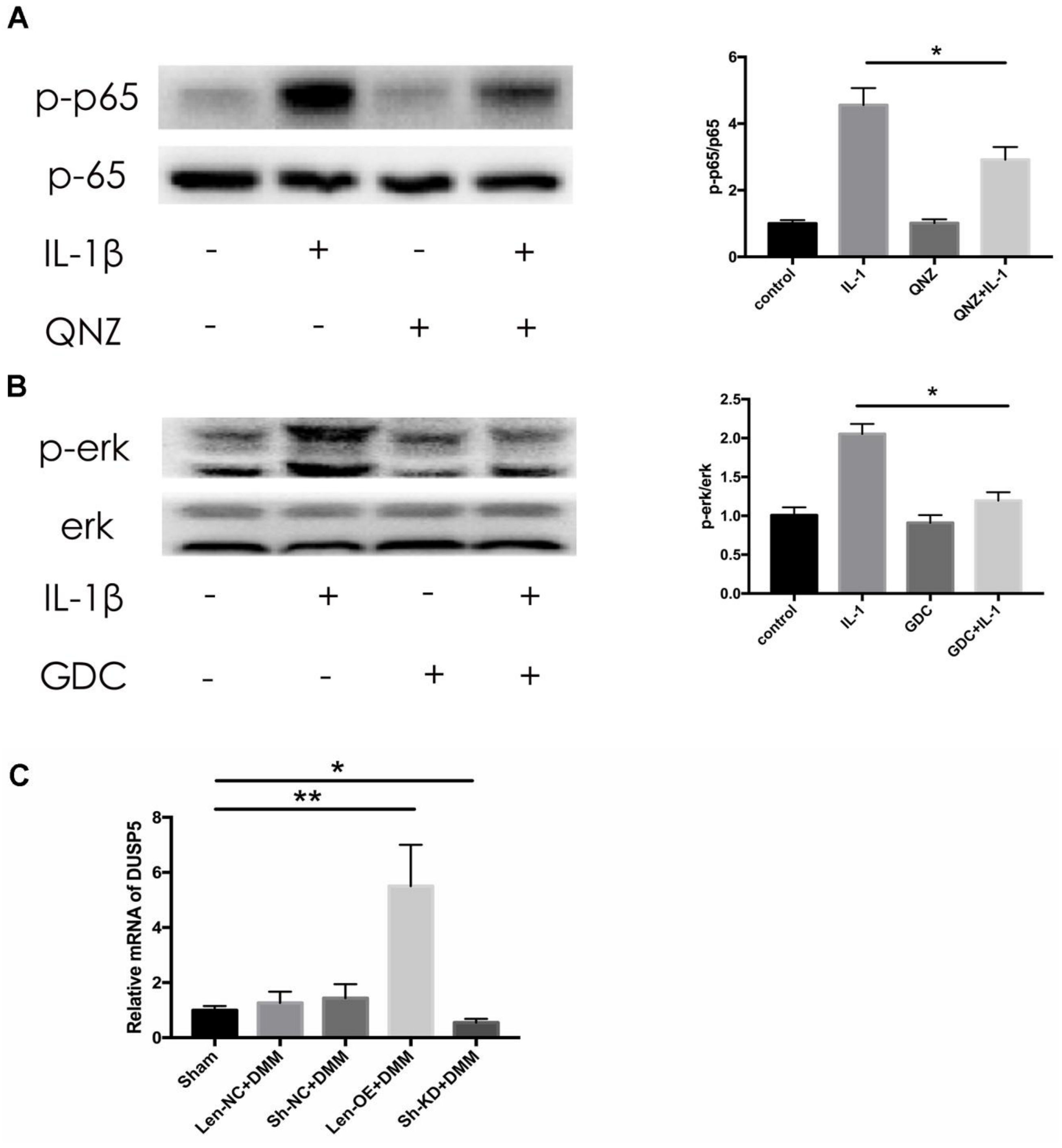

Supplementary Figure 1. (**A**) The effectiveness of inhibitor QNZ. The protein expression and quantitation of p-p65 and p-65. Chondrocytes were treated with 10 μM QNZ for 2 h before incubation with IL-1β (10 ng/mL) for 10 min. (**B**) The effectiveness of inhibitor GDC. The protein expression and quantitation of p-ERK and ERK. Chondrocytes were treated with 6.1 nM GDC for 2 h before incubation with IL-1β (10 ng/mL) for 10 min. (**C**) Levels of DUSP5 were measured by reverse transcription-quantitative polymerase chain reaction. Rats were sacrificed at 8 weeks post-DMM surgery from each group. *$p < 0.05$, **$p<0.01$.
